# Impact of kinetic isotope effects in isotopic studies of metabolic systems

**DOI:** 10.1186/s12918-015-0213-8

**Published:** 2015-09-26

**Authors:** Pierre Millard, Jean-Charles Portais, Pedro Mendes

**Affiliations:** MCISB, Manchester Institute of Biotechnology, University of Manchester, Manchester, UK; School of Computer Science, University of Manchester, Manchester, UK; Université de Toulouse; INSA, UPS, INP; LISBP, Toulouse, France; INRA, UMR792, Ingénierie des Systèmes Biologiques et des Procédés, Toulouse, France; CNRS, UMR5504, Toulouse, France; Center for Quantitative Medicine and Department of Cell Biology, UConn Health, Farmington, Connecticut USA

**Keywords:** Isotope effect, Isotope labeling experiment, Metabolic flux analysis, Fluxomics, Kinetic model, Isotopic model

## Abstract

**Background:**

Isotope labeling experiments (ILEs) are increasingly used to investigate the functioning of metabolic systems. Some enzymes are subject to kinetic isotope effects (KIEs) which modulate reaction rates depending on the isotopic composition of their substrate(s). KIEs may therefore affect both the propagation of isotopes through metabolic networks and their operation, and ultimately jeopardize the biological value of ILEs. However, the actual impact of KIEs on metabolism has never been investigated at the system level.

**Results:**

First, we developed a framework which integrates KIEs into kinetic and isotopic models of metabolism, thereby accounting for their system-wide effects on metabolite concentrations, metabolic fluxes, and isotopic patterns. Then, we applied this framework to assess the impact of KIEs on the central carbon metabolism of *Escherichia coli* in the context of ^13^C-ILEs, under different situations commonly encountered in laboratories. Results showed that the impact of KIEs strongly depends on the label input and on the variable considered but is significantly lower than expected intuitively from measurements on isolated enzymes. The global robustness of both the metabolic operation and isotopic patterns largely emerge from intrinsic properties of metabolic networks, such as the distribution of control across the network and bidirectional isotope exchange.

**Conclusions:**

These results demonstrate the necessity of investigating the impact of KIEs at the level of the entire system, contradict previous hypotheses that KIEs would have a strong effect on isotopic distributions and on flux determination, and strengthen the biological value of ^13^C-ILEs. The proposed modeling framework is generic and can be used to investigate the impact of all the isotopic tracers (^2^H, ^13^C, ^15^N, ^18^O, etc.) on different isotopic datasets and metabolic systems. By allowing the integration of isotopic and metabolomics data collected under stationary and/or non-stationary conditions, it may also assist interpretations of ILEs and facilitate the development of more accurate kinetic models with improved explicative and predictive capabilities.

**Electronic supplementary material:**

The online version of this article (doi:10.1186/s12918-015-0213-8) contains supplementary material, which is available to authorized users.

## Background

Isotopic studies are increasingly used to better understand the organization and the functioning of metabolic systems in systems biology and to assist the design of efficient and robust production strains in biotechnology [1–4]. The basic principle of isotope labeling experiments (ILEs) consists of: *i*) cultivating cells on an isotopically enriched substrate [5], *ii*) collecting metabolites using adequate sampling methods [6–8], and *iii*) quantifying their isotopic content by mass spectrometry (MS) and/or nuclear magnetic resonance (NMR) [9–12]. Isotopic data are typically exploited to identify metabolic pathways [13] and metabolites [14], to assist the discovery of new metabolite-protein regulatory interactions [15], to profile metabolic variants [16, 17], and to quantify metabolite and flux responses to environmental and genetic perturbations [18, 19].

The biological insights obtained from ILEs are valid provided isotopes do not modify the operation of the metabolic network investigated and no isotopic fractionation occurs in the network. However, many enzymes are subject to kinetic isotope effects (KIEs) that modify reaction rates depending on the isotopic composition of their substrate(s) [20]. For instance, it is well established that Rubisco, the carbon fixing enzyme of the Calvin cycle, assimilates preferentially ^12^CO_2_ over ^13^CO_2_ [21]. Therefore, KIEs modify the propagation of isotopes through the network and the isotopic data collected in ILEs. Since KIEs modify reaction rates, one can intuitively expect they also impact the operation of the metabolic systems investigated (*i.e.* metabolite concentrations and fluxes), and thus isotopic data themselves. However, the metabolic control may be distributed over different steps of a pathway, and in this case the change of rate constant of a particular reaction may not directly translate into a change of flux through the corresponding pathway. This systemic property of control distribution across the network was theoretically developed under the framework of metabolic control analysis [22, 23] and has been repeatedly observed *in vivo* [24–27]. Changes of reaction rates may also propagate through the network and affect the metabolic operation and the distribution of isotopes in other parts of the network. Hence, KIEs may exert considerable effects over all variables of the system (metabolite concentrations, fluxes, and distribution of isotopes), and thus potentially jeopardize the biological value of ILEs. However, these effects are usually assumed to be negligible.

The first attempt to assess the impact of KIEs on the distribution of isotopes in intracellular metabolites was published recently, in the context of ^13^C-ILEs [28]. They concluded that in some situations the impact of KIEs of pyruvate dehydrogenase on the labeling of acetyl-CoA may be of comparable size to the measurement errors in GC-MS, hence introducing a significant bias in the estimated fluxes. In another study, Fan *et al.* [19] carried out ^2^H-ILEs to quantify the contribution of the pentose phosphate pathway to NADPH production in *Escherichia coli*. They corrected isotopic measurements for the strong isotopic fractionation related to ^2^H, assuming they have no impact on fluxes. Although highly informative, these studies were carried out at the level of a single metabolic node. Thus, they fail to account for the system-wide effect that these KIEs have over all metabolite concentrations, fluxes and isotopic distributions.

In this study, we first developed a modeling framework which combines isotopic and kinetic models, while taking the system-wide KIEs into account. This framework is based on the fluxomer concept [29], which represents the rates at which metabolic reactions transform each substrate(s) isotopomers. This allows for a straightforward integration of KIEs, which are measured for a particular (subset of) substrate(s) isotopomer(s). This generic framework was applied to investigate the impact of KIEs on the glycolytic and pentose phosphate pathways of the model bacterium *E. coli*, in the context of the most widely used ^13^C-ILEs. The impact of KIEs on metabolite concentrations, fluxes and isotopic distributions was quantified under a broad range of situations commonly encountered in laboratories (in terms of label input, metabolic state, and isotopic information considered).

## Methods

### Model construction

The kinetic model published by [30] was used as a scaffold to develop the isotopic system, as detailed in the results section, with carbon atom transitions taken from [6]. Since reversibility results in bidirectional isotope exchange [31], forward and backward rates of reversible reactions were considered separately by decomposing the rate laws into forward and backward components. For instance, in the case of the phosphoglycerate mutase (GPM) described by a reversible Michaelis-Menten-type rate law:

1 $$ {v}_{gpm}^{net}={V}_{max}\frac{PG3-\frac{PG2}{K_{eq}}}{K_m^{PG3}.\left(1+\frac{PG2}{K_m^{PG2}}\right)+PG3} $$

the forward (*v*^*forw*^) and backward (*v*^*back*^) rates are equal to:

2 $$ {v}_{gpm}^{forw}={V}_{max}\frac{PG3}{K_m^{PG3}.\left(1+\frac{PG2}{K_m^{PG2}}\right)+PG3} $$

3 $$ {v}_{gpm}^{back}={V}_{max}\frac{\frac{PG2}{K_{eq}}}{K_m^{PG3}.\left(1+\frac{PG2}{K_m^{PG2}}\right)+PG3} $$

The model thus contains 58 fluxes (32 irreversible and 13 reversible reactions) decomposed into 7376 fluxomers. This model describes the dynamics of 616 isotopomers (and 94 isotopologues) from 17 metabolites.

Five reactions included in the model are catalyzed by enzymes for which KIEs were identified (Table 1). However, KIEs were measured only for singly labeled isotopomers. KIEs related to multiply labeled isotopomers were approximated assuming effects are cumulative [28]. Multiply labeled isotopomers are therefore more strongly impacted by KIEs than singly labeled isotopomers.

**Table 1:** Kinetic isotope effects measured on five central metabolic enzymes. For multiply labeled isotopomers, α coefficients were approximated from KIEs of singly labeled isotopomers assuming KIEs are cumulative [28]; for instance, *α*_*PDH*_^*PYR*_110^ = *α*_*PDH*_^*PYR*_100^. *α*_*PDH*_^*PYR*_010^

Enzyme	Reaction	Substrate	Isotopomer	α	Ref.
6-phosphogluconate dehydrogenase	GND	PGN	100000	0.9905	[48]
glucose-6-phosphate dehydrogenase	G6PDH	G6P	100000	0.9837	[49]
fructose-1,6-bisphosphate aldolase	ALD	FBP	001000	0.9843	[50]
ribulose-5-phosphate epimerase	RPE	RB5P	01000	0.9931	[51]
			00100	0.9818	
			00010	0.9852	
			00001	0.9980	
pyruvate dehydrogenase	PDH	PYR	100	0.9908	[52]
			010	0.9791	
			001	0.9969	

### Simulations

The system of ordinary differential equations was automatically generated in FORTRAN, and steady-states were calculated using the LSODE solver implemented in the *runsteady* function of the *rootSolve* package (v1.6.5) of R (v3.0, www.r-project.org). Absolute and relative error tolerances were set to 10^−8^ and 10^−6^, respectively. All the scripts used to construct the models, perform the simulations and generate the figures are distributed in supplementary information (Additional file 1) under an open source license to ensure reproducibility and reusability.

### Global sensitivity analysis

A global sensitivity analysis was carried out to evaluate the robustness of our conclusions under a broad range of metabolic states. To this end, steady-states were simulated for 10,000 sets of random enzyme levels generated using the *runif* function of R. For each set, maximum reaction rates (V_max_, which is proportional to the level of active enzyme) were sampled over two orders of magnitude (between 0.1 and 10 times the enzyme levels of the initial model) from a log uniform distribution to ensure each order of magnitude to be sampled in similar proportions.

## Results and discussion

### Modeling isotopic fractionation

**Fig. 1:**
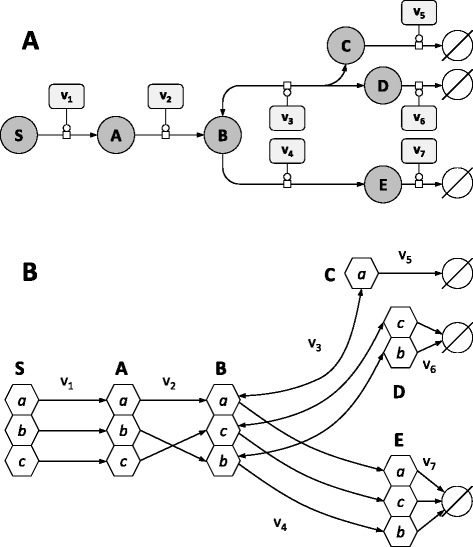
A simple example network. **a** Representation of the example network in Systems Biology Graphical Notation format (SBGN, www.sbgn.org) [47]. Rounded rectangles represent enzymes, circles represent metabolites, and circles with dark bands along their bottoms represent metabolites which appear multiple times in the map. The concentration and labeling state of *S* are fixed, and reactions *v*_5–7_ represent sink reactions. **b** Carbon transition network used to construct the isotopomers balances, where hexagons represent carbon positions. While *v*_1_ and *v*_4_ do not modify the carbon skeleton between substrates and products, other reactions result in carbon inversion (*v*_2_) or in the reversible cleavage of carbon-carbon bonds (*v*_3_)

A simple network is given in Fig. 1a as an example, with the corresponding carbon atom transitions shown in Fig. 1b. The labeling state of a metabolite containing *n* carbon atoms is denoted by the coefficients (*a*, *b*, …, *n*), which take a value of *0* if the atom in the corresponding carbon position is ^12^C or a value of *1* if the atom is ^13^C. For instance, *B*_0,1,1_ represents the isotopomer of the metabolite *B* which is unlabeled in position 1 and labeled in positions 2 and 3. In the absence of KIEs, each isotopomer of a given metabolite reacts with a probability equal to its abundance relative to the total metabolite pool. Each isotopomer *A*_*a,b,c*_ is thus produced at the rate *v*_1_ weighted by ${\overline{S}}_{a,b,c}$, which denotes the concentration of the isotopomer *S*_*a,b,c*_ relative to the total *S* pool (${\displaystyle \sum_{a,b,c}}{S}_{a,b,c}$). Similarly, *A*_*a,b,c*_ is consumed at the rate *v*_2_ weighted by *Ā*_*a*,*b*,*c*_, which denotes the concentration of the isotopomer *A*_*a,b,c*_ relative to the total *A* pool. Therefore, the balance around the isotopomer *A*_*a,b,c*_ is:

4 $$ \frac{d{A}_{a,b,c}}{dt}={v}_1.{\overline{S}}_{a,b,c}-{v}_2.{\overline{A}}_{a,b,c} $$

where *v*_1_ and *v*_2_ are functions of the concentrations of reactants and effectors, and of the kinetic parameters of the enzymes catalyzing these reactions. This equation can be rewritten using the fluxomer variable introduced by [29]. A fluxomer is the rate at which a metabolic reaction transforms one or several substrate(s) isotopomers into product(s) isotopomers. Fluxomers are usually expressed as rate fractions [28, 29], which allows a direct mapping between fluxomers and isotopomer abundances. However, this is not relevant to calculate isotopomers derivatives. Therefore, fluxomers were expressed as absolute rates rather than rate fractions. In the absence of KIEs, the fluxomer *f*_1_^*a*,*b*,*c*^ which consumes *S*_*a,b,c*_ is defined by:

5 $$ {f}_1^{S_{a,b,c}}={v}_1.{\overline{S}}_{a,b,c} $$

and the mass balance around *A*_*a,b,c*_ can be rewritten as:

6 $$ \frac{d{A}_{a,b,c}}{dt}={f}_1^{S_{a,b,c}}-{f}_2^{A_{a,b,c}} $$

It must be noted that the term "fluxomer" is somewhat misleading in the context of this study since it does not define a *flux* (which is a term used for the rate when the reaction is part of a whole system) but rather a *local rate* (*i.e.* the rate when considering the reaction isolated from the rest of the system). Bearing that in mind, we keep this terminology for sake of simplicity, but note the important distinction between these terms.

KIEs affect the rate constant of reactions depending on the isotopic composition of their substrate(s). Consequently, KIEs were included by weighting each fluxomer with a coefficient α which represents the KIE of a given enzyme with respect to its substrate(s) isotopomers. For instance, each coefficient ${\alpha}_{v_2}^{A_{a,b,c}}$ is defined as:

7 $$ {\alpha}_{v_2}^{A_{a,b,c}}=\frac{v_2^{A_{a,b,c}}}{v_2^{A_{0,0,0}}} $$

where ${v}_2^{A_{a,b,c}}$ (${v}_2^{A_{0,0,0}}$) represents the rate of reaction 2 when isotopomer *A*_*a,b,c*_ (*A*_0,0,0_) is its unique substrate. The mass balance around *A*_*a,b,c*_ finally becomes:

8 $$ \frac{d{A}_{a,b,c}}{dt}={\alpha}_{v_1}^{S_{a,b,c}}.{f}_1^{S_{a,b,c}}-{\alpha}_{v_2}^{A_{a,b,c}}.{f}_2^{A_{a,b,c}} $$

To calculate the mass balances for the isotopomers of pool *B*, two additional points must be taken into account. First, it is worth recalling that bidirectional isotope exchange that arise from reversibility significantly impacts the distribution of isotopes through the network [31]. Forward and backward reaction rates must be considered separately in the case of reversible reactions, like *v*_3_ in the model considered here. Second, in the case of reactions with more than one substrate, the impact of KIEs on reaction rate may depend on the isotopic composition of each substrate. Therefore, one fluxomer must be defined for each combination of substrates isotopomers. Reaction *v*_3_ was first decomposed into its forward (*v*_3_^*forw*^) and backward (*v*_3_^*back*^) components, each of them being then decomposed into fluxomers:

9 $$ {f}_{3,\  forw}^{B_{a,b,c}}={v}_3^{forw}.{\overline{B}}_{a,b,c} $$

10 $$ {f}_{3,\  back}^{C_a,{D}_{b,c}}={v}_3^{back}.{\overline{C}}_a.{\overline{D}}_{b,c} $$

and the mass balance around *B*_*a,c,b*_ is:

11 $$ \frac{d{B}_{a,c,b}}{dt}={\alpha}_{v_2}^{A_{a,b,c}}.{f}_2^{A_{a,b,c}}+{\alpha}_{v_3}^{C_a}.{\alpha}_{v_3}^{D_{c,b}}.{f}_{3,\  back}^{C_a,{D}_{c,b}}-{\alpha}_{v_3}^{B_{a,c,b}}.{f}_{3,\  forw}^{B_{a,c,b}}-{\alpha}_{v_4}^{B_{a,c,b}}.{f}_4^{B_{a,c,b}} $$

Using the same formalism, the mass balances around the isotopomers of C, D and E are:

12 $$ \frac{d{C}_a}{dt}={\displaystyle \sum_{b,c}}\left({\alpha}_{v_3}^{B_{a,b,c}}.{f}_{3,\  forw}^{B_{a,b,c}}\right)-{\displaystyle \sum_{b,c}}\left({\alpha}_{v_3}^{C_a}.{\alpha}_{v_3}^{D_{b,c}}.{f}_{3,\  back}^{C_a,{D}_{b,c}}\right)-{\alpha}_{v_5}^{C_a}.{f}_5^{C_a} $$

13 $$ \frac{d{D}_{b,c}}{dt}=\sum_a\left({\alpha}_{v_3}^{B_{a,b,c}}.{f}_{3, forw}^{B_{a,b,c}}\right)-\sum_a\left({\alpha}_{v_3}^{C_a}.{\alpha}_{v_3}^{D_{b,c}}.{f}_{3, back}^{C_a,{D}_{b,c}}\right)-{\alpha}_{v_6}^{D_{b,c}}.{f}_6^{D_{b,c}} $$

14 $$ \frac{d{E}_{a,b,c}}{dt}={\alpha}_{v_4}^{B_{a,b,c}}.{f}_4^{B_{a,b,c}}-{\alpha}_{v_7}^{E_{a,b,c}}.{f}_7^{E_{a,b,c}} $$

**Fig. 2:**
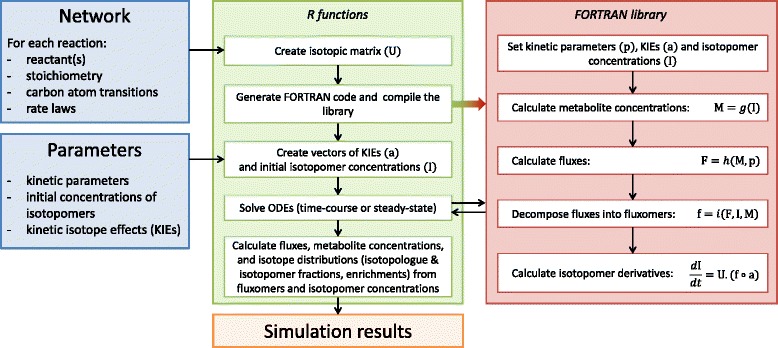
Diagram of the procedure developed to simulate the system-level impact of KIEs on metabolism. Blue boxes represent the input (definition of the metabolic system investigated and of its parameters), green and red boxes represent the steps implemented in R and FORTRAN, respectively, and orange box represents the output (simulation results). R is used to *i*) construct isotopic matrices, *ii*) create the FORTRAN library which contains the ODEs system, *iii*) initialize parameters, *iv*) solve ODEs and *v*) gather the results. Briefly, the FORTRAN library performs the following steps: *i*) computes metabolite concentrations (M) by summing isotopomer concentrations (I), function *g*, *ii*) calculates fluxes from metabolite concentrations (M) and kinetic parameters (p), function *h*, *iii*) decomposes fluxes (F) into fluxomers (f) using concentrations of isotopomers (I) and metabolites (M), function *i*, and *iv*) isotopomers derivatives are finally calculated from the isotopic matrix (U) and the fluxomers (f) and KIEs (a) vectors (Eq. 15)

The system of ordinary differential equations (ODEs) defined by Eqs. 8, 11, 12, 13, 14 can be transformed into matrix notation by defining a matrix *U* where rows correspond to isotopomers and columns correspond to fluxomers. This matrix contains the stoichiometric coefficients of each substrate(s) and product(s) isotopomer(s) for each fluxomer *f*_*r*_^*p*^ (where *p* represents a particular (set of) substrate(s) isotopomer(s) consumed through the reaction *r*). A vector *f* is then defined that contains all the fluxomers (calculated from the kinetic rate laws of each reaction, the concentrations of reactants and effectors, and the relative abundances of substrate(s) isotopomers), and a vector *a* that contains the coefficients *α*_*r*_^*p*^ for each fluxomer *f*_*r*_^*p*^. The isotopomer balances can be calculated by:

15 $$ \frac{dI}{dt}=U.\left(f(t)\circ a\right) $$

where *I* is the vector of isotopomer concentrations and ∘ is the element-wise product operator, *i.e.* (*f*(*t*) ∘ *a*)_*x*_ = *f*_*x*_(*t*). *a*_*x*_. The dynamics (and steady-states) of all the isotopomers can be simulated by solving this system of ODEs. Finally, fluxes and metabolite concentrations can be calculated by summing fluxomers and isotopomer concentrations, respectively. The numerical procedure and its implementation are summarized in Fig. 2.

### Impact of KIEs on the operation of *E. coli* central metabolism in ^13^C-labeling experiments

**Fig. 3:**
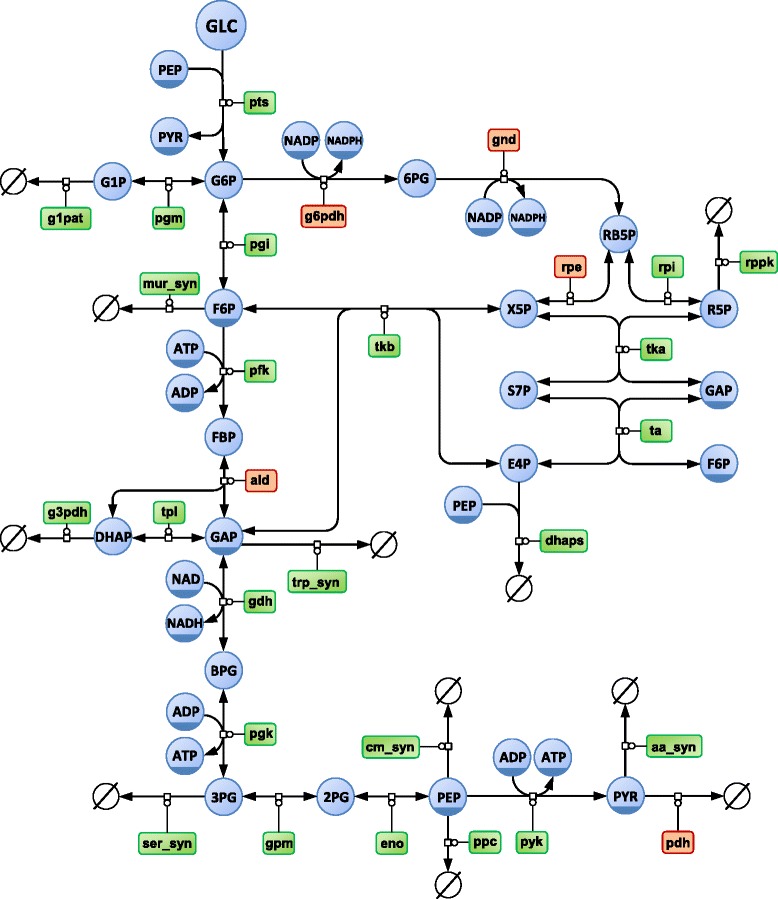
Central carbon metabolic network of *Escherichia coli*. Representation of the glucose uptake, glycolytic and pentose phosphate pathways of *E. coli* in SBGN format. Circles represent metabolites and rounded rectangles represent enzymes. Enzymes subjected to kinetic isotope effects are highlighted in orange. Note that the 17 reactions of dilution of intracellular pools due to growth are omitted from this diagram for clarity of the layout

We applied this framework to evaluate the impact of KIEs on the operation of the glycolytic and pentose phosphate pathways of the model bacterium *Escherichia coli* grown on ^13^C-labeled glucose. The kinetic model published by [30] was used as a scaffold to construct the isotopic model, with the carbon transition network taken from [6] (Fig. 3). KIEs measured on five enzymes were included (glucose-6-phosphate dehydrogenase, G6PDH; 6-phosphogluconate dehydrogenase, GND; ribulose-5-phosphate epimerase, RPE; fructose-1,6-bisphosphate aldolase, ALD; and pyruvate dehydrogenase, PDH), with the corresponding *α*_*r*_^*p*^ coefficients listed in Table 1. Steady-states were simulated for glucose at natural abundance and six different glucose labelings: U-^13^C-glucose, a mixture containing 80 % of 1-^13^C- and 20 % of U-^13^C-glucose, an equimolar mixture of ^12^C- and U-^13^C-glucose, 2,3,4,5,6-^13^C-glucose, 1,2-^13^C-glucose and 3,4-^13^C-glucose. These label inputs are representative of those commonly used in ^13^C-ILEs [6, 32–35].

**Fig. 4:**
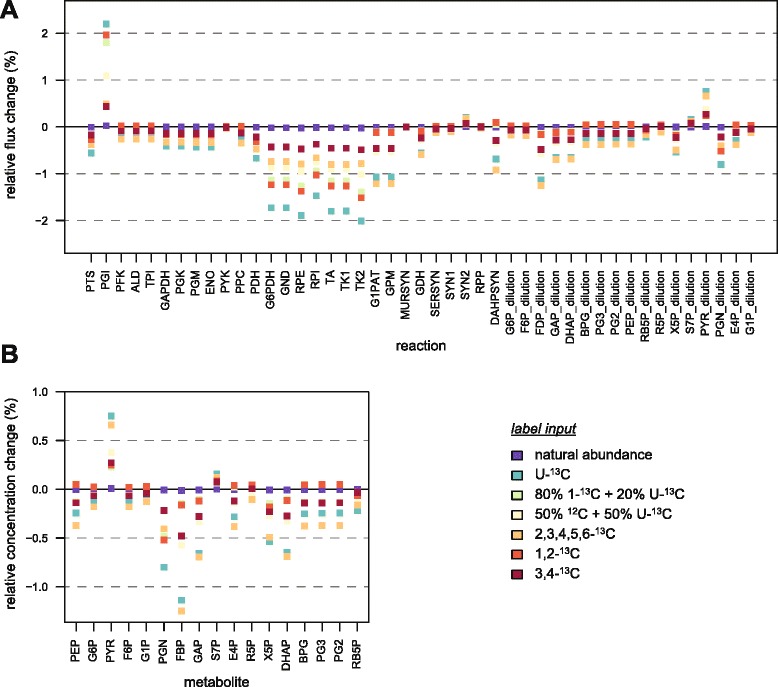
Impact of KIEs on the operation of *E. coli* central carbon metabolism. The impact of KIEs on the (steady-state) fluxes (**a**) and metabolite concentrations (**b**) of *E. coli* central carbon metabolism was simulated for glucose at natural abundance and six label inputs commonly used in ^13^C-labeling experiments. Impact of KIEs was defined as the relative difference between steady-states reached with and without KIEs, *i.e.* with alpha coefficients set to their experimental values (Table 1) or to 1, respectively

For the metabolic state represented by the model, the relative changes of fluxes and metabolite concentrations caused by KIEs for each glucose input are shown in Fig. 4a and b, respectively. Impact on these variables is low (<2 %), with maximal changes observed when cells are grown on U-^13^C-glucose. Since in this situation all the metabolites are fully labeled, impact of KIEs on reaction rates is also maximal. For instance, assuming that KIEs are cumulative at the level of a single reaction [28], the reaction rate of the ribulose-5-phosphate epimerase (RPE) is reduced by 4.14 % when RB5P is fully labeled. However, the predicted change of flux (−1.7 %) is lower than that, which can be explained by the distribution of flux control over several reactions of the network. Moreover, the signs of the changes of fluxes and metabolite concentrations depend on the label input. This indicates that the impact of KIEs on the system cannot be intuitively inferred from their impact at the reaction level, even qualitatively. The impact of KIEs must be investigated at the system-level and not at the level of an isolated reaction or metabolic node.

The predicted changes of metabolite concentrations are significantly lower than the precision commonly reached in metabolomics (*circa* 10 %) [18, 36], hence they cannot be detected with current methods. The same situation is observed for fluxes, with changes caused by KIEs slightly lower than the flux precision commonly obtained in ^13^C-metabolic flux analysis (^13^C-MFA) [5, 6, 12]. However, the predicted flux changes caused by KIEs are one order of magnitude higher than the flux precision estimated using parallel labeling experiments with different label inputs [37]. Therefore, fluxes obtained in high-resolution ^13^C-MFA studies might be biased, and KIEs should be considered during flux estimation and sensitivity analysis when such high degree of precision can be reached.

### Impact of KIEs on isotopic patterns of central metabolites

**Fig. 5:**
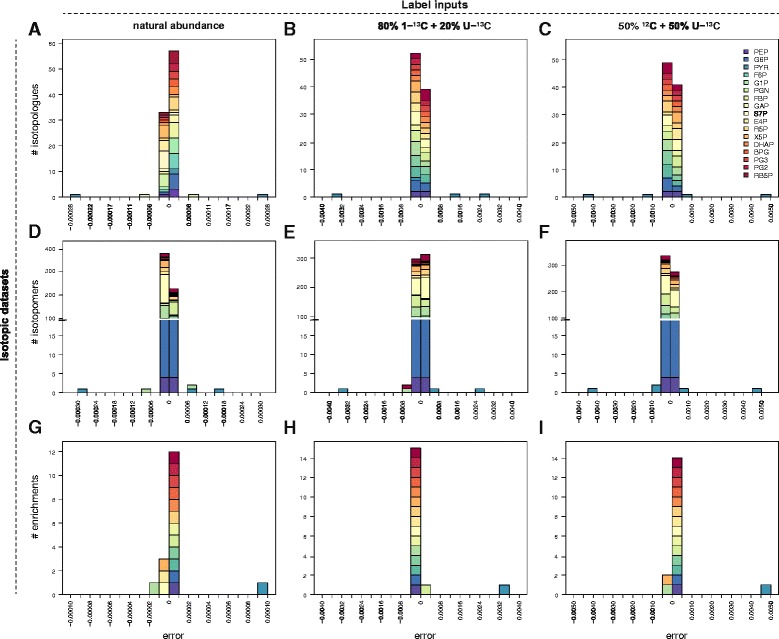
Impact of KIEs on the distribution of isotopes in intracellular metabolites. The impact of KIEs on the different isotopic datasets of central metabolites (isotopologue abundances in panels **a**-**c**, isotopomer abundances in panels **d**-**f** and enrichments in panels **g**-**i**) was simulated for three label inputs: glucose at natural abundance, 80 % 1-^13^C-glucose + 20 % U-^13^C-glucose, and 50 % ^12^C-glucose + 50 % U-^13^C-glucose. Error is defined as the absolute difference between isotopic data simulated with and without KIEs, *i.e.* with alpha coefficients set to their experimental values (Table 1) or to 1, respectively

Isotopic data collected in ^13^C-ILEs can be exploited quantitatively to infer information on the structure and the operation of metabolic systems. Since KIEs may impact isotopic data and ultimately jeopardize the validity of the biological interpretations, their impact must be assessed rigorously. This was performed for the various isotopic data that can be acquired with modern analytical platforms: isotopomer distributions [11, 38, 39], isotopologue distributions [7, 40] and molecular enrichments [40, 41]. The corresponding (steady-state) isotopic datasets were simulated in the absence or presence of KIEs, for three different label inputs: glucose at natural abundance, a mixture containing 80 % of 1-^13^C-glucose and 20 % of U-^13^C-glucose, and an equimolar mixture of ^12^C- and U-^13^C-glucose. The distributions of errors caused by KIEs are shown in Fig. 5 for each isotopic dataset and label input.

The sensitivity of isotopic data to KIEs strongly depends on the isotopic information considered and on the label input. As a general trend, the impact of KIEs is maximal for the equimolar mixture of ^12^C- and U-^13^C-glucose, and minimal for glucose at natural abundance. The predicted errors caused by KIEs are surprisingly low – below 0.001 – for virtually all metabolites and isotopic data. The only exception is pyruvate, with predicted errors up to 0.0045 for its isotopologues, 0.0047 for its isotopomers, and 0.0045 for its molecular enrichment. These errors originate exclusively from KIEs of pyruvate dehydrogenase (PDH). Here again, the system-level impact of KIEs on the isotopic distributions strongly differs from the impact that can be expected for isolated reactions. Moreover, impacts of individual KIEs do not add up at the system level, contrary to what was previously hypothesized [28].

This example illustrates how the framework developed here can be used to evaluate the sensitivity of each isotopic data to KIEs. The less reliable measurements with respect to KIEs can be identified and discarded from the datasets exploited in quantitative approaches such as ^13^C-MFA. In the situations investigated here, the biases caused by KIEs on the distribution of isotopes into the glycolytic and pentose phosphate pathway metabolic intermediates are significantly lower than the precision and accuracy obtained by MS (or even MS/MS) [40, 42] and NMR [10], which is around 1 %, and cannot be detected. Many other central metabolic enzymes are likely subject to KIEs, which may significantly increase their predicted impact on isotopic patterns. However, the present results support the assumption that KIEs can be neglected when studies focus on the glycolytic and pentose phosphate pathways and are based on isotopic information obtained from metabolic intermediates.

### Isotopic data are robust to KIEs under a broad range of metabolic states

**Fig. 6:**
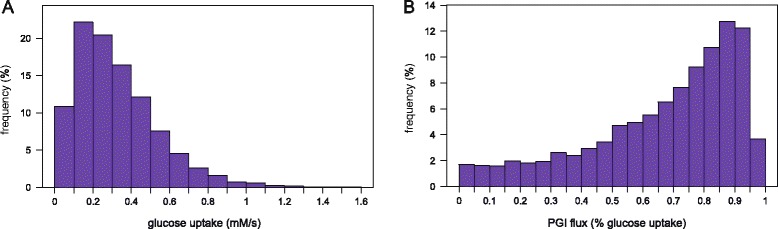
Random sampling of enzyme levels results in a broad range of metabolic states. Distribution of the steady-state glucose uptake (**a**) and glycolytic (**b**) fluxes simulated from 10,000 sets of random enzyme levels

**Fig. 7:**
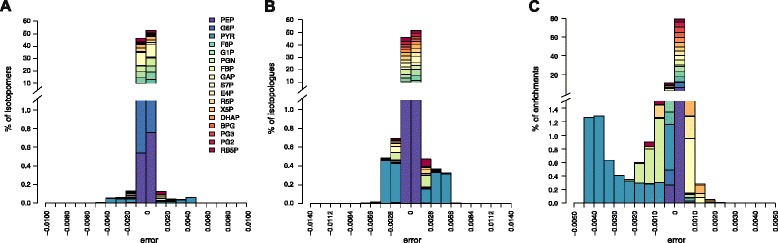
Global impact of KIEs on isotopic patterns. Distributions of errors caused by KIEs on the isotopomer abundances (**a**), isotopologue abundances (**b**) and enrichments (**c**) of metabolic intermediates, simulated from 10,000 sets of random enzyme levels to cover a broad range of metabolic states. Error is defined as the absolute difference between isotopic data simulated with and without KIEs, *i.e.* with alpha coefficients set to their experimental values (Table 1) or to 1, respectively

**Fig. 8:**
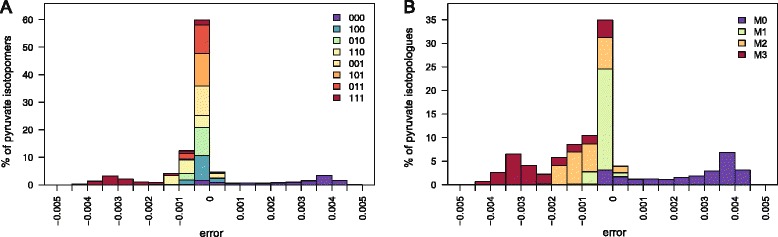
Global impact of KIEs on the isotopic content of pyruvate. The distributions of errors caused by KIEs on the (steady-state) abundances of pyruvate isotopomers (**a**) and isotopologues (**b**) were estimated from 10,000 sets of random enzyme levels. Error is defined as the absolute difference between isotopic data simulated with and without KIEs, *i.e.* with alpha coefficients set to their experimental values (Table 1) or to 1, respectively

The above results indicate that isotopic data are robust to KIEs locally, *i.e.* near the given metabolic state investigated. However, metabolic systems are highly non-linear and the impact of KIEs might strongly differ depending on the metabolic state considered [28]. To grasp the global impact of KIEs on isotopic distributions, we first generated 10,000 sets of random enzyme levels for which we calculated the steady-states of the system with 80 % 1-^13^C- glucose + 20 % U-^13^C-glucose as label input. Although it is clear that these sets are not representative of those expressed *in vivo*, they are expected to result in different metabolic states. This was confirmed by analyzing the distribution of some systemic steady-state variables, *e.g.* the glucose uptake flux varied between 0.02 and 1.6 mmol/g_DW_/s, and the carbon channeled into glycolysis varied between 0 and 98 % of the glucose uptake flux (Fig. 6). For each metabolic state, errors caused by KIEs on the three isotopic datasets were calculated and are summarized in Fig. 7. Errors remain very low for the vast majority of isotopic data, with 99.7 % of absolute isotopomer errors below 0.002, 99.0 % of absolute isotopologue errors below 0.003, and 97 % of enrichment errors below 0.003. Here again errors strongly depend on the metabolite considered. For all isotopic datasets, the highest errors are related to pyruvate, consistently with the above results. Still, errors for this metabolite errors are minor in most cases (<0.003 for 89 % of its isotopomers and 77 % of its isotopologues). A more detailed analysis revealed that only the proportions of the unlabeled and fully labeled pyruvate isotopomers (and isotopologues) are significantly impacted by KIEs (Fig. 8), errors regarding other isotopomers (and isotopologues) remain very low (<0.002) under all the metabolic states. Errors related to other metabolites are minor (<0.003) for all the datasets, for instance the highest absolute error for glyceraldehyde-3-phosphate (GAP) isotopologues is 0.002. These results highlight a global robustness of the isotopic data measured on glycolytic and pentose phosphate pathway intermediates, and soften the conclusions of [28].

### Reversibility is a key determinant of the robustness of isotopic patterns

**Fig. 9:**
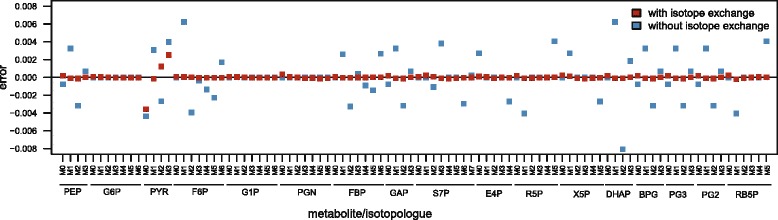
Impact of bidirectional isotope exchanges on the robustness of isotopic data to KIEs. The impact of KIEs on the isotopologue abundances of central metabolites was simulated for 80 % 1-^13^C-glucose + 20 % U-^13^C-glucose as label input, with (red squares) or without (blue squares) considering isotope exchange caused by reaction reversibility. Error is defined as the absolute difference between isotopic data simulated with and without KIEs, *i.e.* with alpha coefficients set to their experimental values (Table 1) or to 1, respectively

In a linear pathway with an irreversible step (*i.e.* not subject to feedback inhibition by its product(s)), reactions downstream the irreversible step do not exert any flux control [43]. Reversibility therefore participates in the robustness of the metabolic operation to KIEs by contributing to the distribution of (flux and concentration) control via feedback regulation. In ILEs, another consequence of reversibility is the bidirectional exchange of isotopes that occurs between substrates and products of reversible reactions. To test if this exchange also plays a role in the robustness of isotope distribution to KIEs, a model of the same network was generated without explicitly considering isotope exchange in the isotopomer balances, *i.e*. without decoupling forward and backward reaction rates when constructing the equation system. It is important to mention that all the systemic properties that emerge from metabolic regulation (in particular the distributed control) are identical in both models since the rate laws remain unchanged. With the two models, the impact of KIEs on isotopologue abundances was simulated with and without isotope exchange (Fig. 9). Errors caused by KIEs were significantly higher for all metabolites when exchange was not explicitly considered, with a 10-fold increase of the mean of absolute errors (0.0014 vs 0.00015). Moreover, errors increased for all metabolites. For instance, while errors on fructose-6-phosphate (F6P) and dihydroxyacetone phosphate (DHAP) isotopologues are negligible with exchange (<0.0002 for both metabolites), they increase up to 0.006 for F6P and 0.008 for DHAP without exchange. Hence, reversibility appears as a key determinant of the robustness of isotopic data to KIEs, both by contributing to the distribution of control across the network and by enabling bidirectional exchange of isotopes between metabolites.

This is consistent with the ^13^C-depletion observed in amino acids [44] since they are produced by less reversible biosynthetic pathways and are therefore expected to be more sensitive to KIEs. Conversely, no major isotopic fractionation was detected on central metabolites in a recent study aiming to produce standard samples that contain metabolites with controlled and predictable isotopic contents [40]. Our simulations support these conclusions and strengthen the reliability of the proposed strategy. These results stress the necessity to take into account reversibility, or at least to evaluate the potential consequences of neglecting this important property of metabolic systems, when integrating isotopic data into quantitative models.

## Conclusion

In this work we introduce a framework that allows comprehensive and rigorous investigations on the impact of KIEs in isotopic studies of metabolism. This framework integrates KIEs into kinetic and isotopic models of metabolism, thereby accounting for their effects on metabolite concentrations, isotopic patterns, and metabolic fluxes on a system-wide basis.

As a case study, this framework was applied to assess the impact of KIEs on *i*) the operation of the central metabolic network of *Escherichia coli* and *ii*) the distribution of isotopes into metabolic intermediates, in the context of ^13^C-ILEs under metabolic steady state conditions. Simulations showed that the impact of KIEs is lower than expected from measurements obtained on isolated enzymes. Robustness of the metabolic operation – and thus also of the distribution of isotopes – to KIEs partly originates from the distribution of concentration and flux control over several steps of the network. Bidirectional isotope exchange due to reversibility is also a major determinant of the robustness of the isotopic patterns. This robustness was observed for virtually all the metabolic intermediates, for all the label inputs tested, and under a broad range of metabolic states. It is therefore unlikely that KIEs jeopardize the biological insights obtained in ^13^C-ILEs, at least for the metabolic network investigated here. The low impact of KIEs on central metabolic intermediates also confirms the interest of quantifying the labeling directly in these intermediates rather than in metabolic end products (such as proteinogenic amino acids). It is important to note that the impact of KIEs may be significant in other situations (in terms of isotopic tracers, metabolic pathways or analytical platforms) and should be evaluated in the specific context of each study.

These findings soften the suggestions of previous studies that ^13^C- KIEs would have a strong effect on isotopic distributions [28] and stresses the necessity of considering reversibility in isotopic studies. It now appears evident that the impact of KIEs on metabolic systems must be investigated at the system level. Unfortunately, KIEs are still unknown for many enzymes, and in most cases they are measured only for some isotopomers. It seems therefore important to dedicate more efforts to *in vitro* characterization of KIEs. Future knowledge can be readily integrated into the model developed here to obtain a more accurate picture of their impact on the system.

The proposed modeling framework is generic and is applicable for other metabolic systems, for different isotopic tracers (*e.g.*^2^H, ^18^O or ^15^N), or for other systemic variables. It may therefore facilitate accurate interpretation of isotopic data collected in ILEs and increase the reliability of the biological insights inferred from these data. We are also considering the application of this framework to better exploit the rich isotopic information collected in ILEs. In particular, it may allow a more accurate estimation of *in vivo* kinetic parameters of enzymes by integrating isotopic and metabolomic measurements obtained under metabolic and/or isotopic non-stationary states [45]. This is expected to improve the predictive and explicative capabilities of kinetic models, and thereby their applicability for *in silico* design of industrially competitive cell factories [46].

### Availability of supporting data

The data sets supporting the results of this article are included within the article and its additional files.

## Additional file

Additional file 1:
**R scripts used to construct the models, perform the simulations and generate the figures.** (TAR 137 kb)

## Abbreviations

^13^C-MFA: ^13^C-metabolic flux analysis
ALD: Fructose-1,6-bisphosphate aldolase
DHAP: Dihydroxyacetone phosphate
F6P: Fructose-6-phoshate
G6PDH: Glucose-6-phosphate dehydrogenase
GAP: Glyceraldehyde-3-phosphate
GND: 6-phosphogluconate dehydrogenase
ILE: Isotope labeling experiment
KIE: Kinetic isotope effect
MS: Mass spectrometry
NMR: Nuclear magnetic resonance
ODEs: Ordinary differential equations
PDH: Pyruvate dehydrogenase
RPE: Ribulose-5-phosphate epimerase
